# 3D Disease Modelling of Hard and Soft Cancer Using PHA-Based Scaffolds

**DOI:** 10.3390/cancers14143549

**Published:** 2022-07-21

**Authors:** Akanksha Tomar, Pinar Uysal-Onganer, Pooja Basnett, Uttam Pati, Ipsita Roy

**Affiliations:** 1 School of Biotechnology, Jawaharlal Nehru University, New Delhi 110067, India; aakansha.tomar103@gmail.com; 2 Cancer Research Group, School of Life Sciences, College of Liberal Arts and Sciences, University of Westminster, London W1W 6UW, UK; p.onganer@westminster.ac.uk; 3 School of Life Sciences, College of Liberal Arts and Sciences, University of Westminster, London W1W 6XH, UK; p.basnett@westminster.ac.uk; 4 Department of Materials Science and Engineering, Faculty of Engineering, University of Sheffield, Sheffield S10 2TN, UK

**Keywords:** tumour modelling, polyhydroxyalkanoates (PHAs), scaffold, breast cancer, colon cancer, epithelial-mesenchymal transition (EMT)

## Abstract

**Simple Summary:**

Tumour progression in vivo was able to be well mimicked in 3D culture by utilizing biodegradable 10 mm × 10 mm × 8 mm P(3HO-*co*-3HD) and P(3HB)-based 3D scaffolds with a pore size of 30 to 300 µm. Both hard (MCF7 and MDA-MB-231) and soft (HCT116) tumour-related cells were successfully grown on the scaffolds, and their growth patterns were studied for 5 days. MDA-MB-231 tend to grow in clusters, and MCF7 cells form an evenly dispersed layer, which covered most of the 3D PHA scaffolds, while HCT116 formed large colonies within the pockets of the 3D PHA scaffold. Epithelial mesenchymal transition (EMT) marker genes, including *Wnt-11*, *E-cadherin*, *Vim* and *Snail* expression profiles, were like those seen in real tumour samples, which confirmed that the cancer models were exhibiting real tumour-like characteristics with high fidelity. These models are important in mimicking hypoxic tumours and in studying gene expression, cellular signalling, angiogenesis and drug response for translational research.

**Abstract:**

Tumour cells are shown to change shape and lose polarity when they are cultured in 3D, a feature typically associated with tumour progression in vivo, thus making it significant to study cancer cells in an environment that mimics the in vivo milieu. In this study we established hard (MCF7 and MDA-MB-231, breast cancer) and soft (HCT116, colon cancer) 3D cancer tumour models utilizing a blend of P(3HO-*co*-3HD) and P(3HB). P(3HO-*co*-3HD) and P(3HB) belong to a group of natural biodegradable polyesters, PHAs, that are synthesised by microorganisms. The 3D PHA scaffolds produced, with a pore size of 30 to 300 µm, allow for nutrients to diffuse within the scaffold and provide the cells with the flexibility to distribute evenly within the scaffold and grow within the pores. Interestingly, by Day 5, MDA-MB-231 showed dispersed growth in clusters, and MCF7 cells formed an evenly dispersed dense layer, while HCT116 formed large colonies within the pockets of the 3D PHA scaffolds. Our results show Epithelial Mesenchymal Transition (EMT) marker gene expression profiles in the hard tumour cancer models. In the 3D-based PHA scaffolds, MDA-MB-231 cells expressed higher levels of *Wnt-11* and mesenchymal markers, such as *Snail* and its downstream gene *Vim* mRNAs, while MCF7 cells exhibited no change in their expression. On the other hand, MCF7 cells exhibited a significantly increased *E-Cadherin* expression as compared to MDA-MB-231 cells. The expression levels of EMT markers were comparative to their expression reported in the tumour samples, making them good representative of cancer models. In future these models will be helpful in mimicking hypoxic tumours, in studying gene expression, cellular signalling, angiogenesis and drug response more accurately than 2D and perhaps other 3D models.

## 1. Introduction

Cancer remains a major cause of death worldwide with 19.3 million new cases and 10 million deaths worldwide in 2020. The most prevalent types of diagnosed cancers include breast (11% of the total cases), lung (11.4%) and colorectal (10%) cancer, followed by prostrate (7.3%) cancer [1]. Cancers that begin in epithelial cells of glandular tissues and produce fluids and mucous are termed as adenocarcinomas. Most cancers of the breast, colon and prostate are adenocarcinomas. Invasive ductal carcinoma is the most common breast adenocarcinoma. Tissue engineered models are required to provide the cells with a mimic of their native environment while preserving their phenotypes, genotypes and behaviour, in order to provide a high-throughput analysis and cost-effective drug screening [2,3].

Two-dimensional (2D) monolayer cultures have been used to study tumour biology where cells are cultured on rigid materials like glass and polystyrene with no contribution from the extracellular matrix. A growing number of studies recognise the limitations of such 2D cultures for in vitro studies [4,5,6,7,8,9]. Apart from the simplicity and low cost, this method does not entirely reflect the essential physiology of real tissues. The cells are forced into polarity and a flattened shape, and modified mechanical and biochemical signals affect cell–cell communication [7,10,11]. As a result, most 2D studies of cellular network functions do not translate to the in vivo models, thereby hindering the development of effective therapies that can be successfully translated into the clinic. The development of 3D cell culture provides better models for drug screening, translational research and cancer prediction. In animal models, cancer is induced by genetic modification or is surgically implanted into mice. However, the success rates of establishing and propagating theses human solid tumours range from 20% to 50% [12]; in genetically engineered mouse models, the tumours fail to grow synchronously and make comparisons of drug responses difficult, and human versus murine differences further add up to the dissimilarities from the human tumours [13].

In vivo cancer-associated stroma is a three-dimensional (3D) structure consisting of neighbouring cells, extracellular matrix (ECM), blood vessels, immune cells and cytokines. Cancer cells interact with their microenvironment during proliferation, metastasis and during chemo- or radiotherapy [14,15,16,17,18,19,20,21,22]. Therefore, it is important to study cancer cells in an environment that mimics the in vivo milieu. The tumour microenvironment has been investigated extensively [23,24,25,26,27,28,29], nevertheless tumour cell biology in a three-dimensional (3D) environment remains poorly understood. Still, 3D polymer-based cancer models can provide several advantages when compared to animal models, such as reproducibility, complexity in terms of cell types, substrate chemistry, topography, tailored mechanical properties, engineered gas diffusion gradients and ethical sustainability [30].

Several 3D in vitro culture models have been proposed to mimic physiological conditions in the tumour, recreating cell-to-cell contact, tumour cell microenvironment and generating hypoxic-necrotic areas [31]. Studies reveal that various cancer cell lines grown in 2D and 3D cell cultures show differences in cancer-related pathways like mTOR-AKT-S6K (mechanistic target of rapamycin (mTOR)-ribosomal S6 kinase (S6K) pathway), and also vary in their drug response, thus making it important to switch to 3D models [32,33,34]. mTOR is an essential regulator of cell homeostasis including protein translation, glucose and lipid metabolism, as well as cell survival and autophagy, and it is a central player that senses and responds to various extracellular growth signals [35]. Scaffold-free cell culture models do not utilise exogenous artificial platforms for promoting cell growth. Such cellular aggregates, termed as multicellular tumour spheroids (MCTS), are the most popular 3D cell culture method to mimic the tumour microenvironment and the cells produce their own ECM. These only partially recapitulate the microenvironment cues and are difficult to optimise for inconsistencies in their formation. They are often applied to mimic structures of breast cancer, epithelial cancer and endothelial cell angiogenesis. Breast and ovarian cancer cell lines have been studied using Matrigel in multi-well or trans-well plates for gene expression, cellular signal pathways, angiogenesis and chemotherapy response [22,36,37,38,39]. In addition, different types of scaffolds, ranging from non-woven fibre ECM-derived materials to polymers in the form of foams and hydrogels, have been investigated. For developing effective scaffolds it is important that the biomaterial should meet the requirement of the physical properties and biocompatibility. Natural biodegradable polymers are attractive because they are highly biocompatible and hence may be used to support cell growth in vitro and tissue growth in vivo. They are composed of polysaccharides (amylose, cellulose, alginate, chitosan or hyaluronic acid), proteins (collagen or gelatine), nucleic acids or polyhydroxyalkanoates [40]. Alginate is a popular biomaterial for cellular encapsulation and has been used to produce fibres or scaffolds for the 3D culture of cancer cells. Low concentrations of alginate with other hydrogels including gelatine, agarose and gelatine methacrylate (GelMA) have been used to design networks of moderate stiffness and retain biological characteristics, tumorigenicity, metastatic ability and increased drug resistance [41,42]. Gene expression analysis of tumour cells cultured in 2D versus 3D alginate-based, oxygen-controlled tumour models revealed striking interdependence between culture dimensionality and hypoxia response [43]. Gelatine hydrogel microspheres (GM) incorporated into cell aggregates tackle the problem of low oxygen and nutrient supply and demonstrate longer cell viability [44]. Cancer invasion models based on drug-incorporated gelatine microspheres along with cancer cells and cancer-associated fibroblasts are efficient tools of drug screening [45]. Chitosan is another biomaterial known for its biocompatibility, biodegradability, low immunogenicity and low cost. Deacetylated chitosan scaffolds show better attachment of cancer cell lines, and cells grow as three-dimensional clumps on the chitosan matrix [46]. Chitosan forms electrostatic interactions with amine groups of alginate and blends to form an interconnected and porous 3D structure with mechanical strength and shape maintenance, which is significantly improved as compared to neat chitosan [47]. A blend of the hydrogel form of chitosan and hyaluronic acid has been used as a non-adhesive material for spheroids formation [48].

The formation of a solid tumour is central to the development of most kinds of cancer. Solid tumours involve cancers of the ovary, breast, colon, brain and other tissues [49]. Most solid tumours have a complex 3D architecture with different populations of abnormal cells divided into parenchymal and stromal compartments. The interactions between the tumour and the microenvironment result in complexity and heterogeneity of tumours leading to resistance to chemotherapy [27]. The stiffness and fibrosis increase from healthy to malignant tissues and are accompanied with chemoresistance [50]. The stiffness of fibroadenoma (solid, smooth, firm non-cancerous benign lumps), measured by its Young’s modulus value is 11.42 kPa, and that of invasive ductal carcinoma (cancer that begin growing in a milk duct and then invade the fibrous or fatty tissue of the breast outside of the duct) is 22.55 kPa [51]. However, the Young’s modulus of colorectal cancer tissue is 7.51 kPa [52]. These values indicate that breast cancer tissues display higher tissue stiffness than colorectal cancer and are hence the former are referred to as hard tumours and the latter as soft tumours.

Hence, a 3D model for cancer with high fidelity needs to meet many criteria including shape, suitable dimensions, adequate interconnected porosity and suitable mechanical properties to mimic the exact tumour environment. Such a model structure thus needs to be made of a material that is processable into porous 3D structures with tuneable stiffness. Polyhydroxyalkanoates (PHAs) comprise such a family of biodegradable polyesters that are produced by bacterial fermentation under nutrient-limiting conditions [53]. There are two types of PHAs, short chain length PHAs (monomer chain length C_4_-C_5_), or SCL-PHAs, and medium chain length PHAs (monomer chain length C_6_-C_16_), or MCL-PHAs. SCL-PHAs are normally hard and brittle, whereas MCL-PHAs are highly elastomeric in nature. In addition, PHAs are highly biocompatible in nature and exhibit surface properties that allow for the attachment and proliferation of mammalian cells [54,55,56]. The first PHA to be identified was poly(3-hydroxybutyric acid) (P(3HB)), which is a homopolymer of 3-hydroxybutyrate (HB). P(3HB) is a typical SCL-PHA, i.e., a stiff polymer. Examples of MCL-PHAs, the elastomeric member of this family, include Poly(3-hydroxyoctanoate), or P(3HO), and Poly(3-hydoxyoctanoate-co-3-hydroxydecanoate), or P(3HO-*co*-3HD) [57]. Blending is an effective way of developing new polymeric material with tailored mechanical properties and in some cases also leads to improved biocompatibility as compared to the parent components. For example, the viability of mouse fibroblasts (cell line L929) on Poly(3-hydroxybutyrate) films increased significantly upon blending with poly(3-hydroxybutyrate-*co*-3-hydroxyhexanoate), P(3HB-*co*-3HHx) [58,59].

Verification of the biomimetic properties of a 3D cancer model can be carried out using a range of methodologies, and quantification of a suitable marker is one of the best strategies. Aberrant Wnt signalling is a hallmark for many cancers and upregulated Wnt-11 expression is reported in breast cancer [60]. Moreover, it has been established that Wnt-11 expression triggers oestrogen receptor alpha and modulates cellular migration in breast cancer [61]. Wnt-11 is downstream of TGF-β—shown previously to be one of the triggers of epithelial-mesenchymal transition (EMT) and chemoresistance [62,63]. EMT is associated with disruption of intracellular tight junctions and loss of cell–cell contact; during cancer progression it is an accepted phenomenon that is due to the loss of epithelial features and gain of mesenchymal morphology [64]. Wnt-11 would thus indeed be a suitable biomarker to validate the 3D cancer models. EMT is a process of cellular reprogramming of epithelial cells into modulating their cell–cell adhesion properties and gaining mesenchymal characteristics such as increased motility and invasiveness linked to metastasis [65]. The EMT programme is orchestrated through transcription factors like Snail, Slug and Zeb1 that are responsible for the gain of mesenchymal properties [66]. Snail is linked to tumour progression and invasiveness due to its ability to alter the expression of the *Vimentin* gene (*vim)* [67]. The latter is one of the mesenchymal markers responsible for maintaining cell shape, cytoplasm integrity and stabilizing cytoskeletal interactions and is found downstream of the Snail gene. An intercellular adhesion protein, E cadherin, displays the gain of epithelial properties and is inversely correlated to invasion of surrounding tissues and metastasis [68]. In the present study, we mainly focus on altered expression of four genes associated with EMT in the human adenocarcinoma cell line.

In this study, we have established, for the first time, 3D cancer models based on the novel family of biocompatible natural polymers, or PHAs. A blend of the MCL-PHA, P(3HO-*co*-3HD), an elastomeric polymer, and a SCL-PHA, P(3HB), a stiff polymer, was used to tailor the mechanical property of the model. Porous 3D PHA scaffolds were generated with variable pore size, allowing for efficient infiltration of the cells and the required nutrients. The growth pattern of the cell lines representing both hard and soft cancer, i.e., breast cancer (MCF7, MDA-MB-231) and colon cancer (HCT116), respectively, have been investigated within these 3D PHA scaffolds. Two different breast cancer cell lines exhibiting variable growth kinetics were used to compare their temporal growth pattern. While MCF-7 are epithelial-like cells associated with a weak invasiveness and good prognosis, MDA-MB-231 are enriched for epithelial to mesenchymal transition (EMT) markers and possess higher phenotypic plasticity and a more invasive behaviour connected to aggressive disease [69]. The size of the 3D tumour models was developed mimicking their real dimensions in patients. Finally, the expression of EMT markers were quantified within these novel tumour models. The unique PHA-based cancer models developed in this work are an excellent step forward in the provision of tailorable 3D models for the in-depth understanding of cancer progression and therapy.

## 2. Materials and Methods

### 2.1. Bacterial Strains and Chemicals Used

P(3HO-*co*-3HD) was produced using *Pseudomonas mendocina* CH50, which was obtained from the National Collection of Industrial and Marine Bacteria (NCIMB 10541)**,** Aberdeen, UK. P(3HB) was produced using *Bacillus subtilis* OK2, which was obtained from the University of Westminster culture collection. The chemicals used for the production and characterisation of PHAs were purchased from Sigma-Aldrich or BDH Ltd. (Dorset, UK), VWR (Leicestershire, UK), unless otherwise stated.

### 2.2. Production and Extraction of P(3HB) and P(3HO-co-3HD)

P(3HB) was produced by *Bacillus subtilis* OK2 using glucose at 35 g/L concentration as the sole carbon substrate. The sterile nutrient broth was inoculated with a single colony of *Bacillus subtilis* OK2 and incubated for 16 h at 30 °C, 200 rpm. Then, 10% (*v*/*v*) of the inoculum was used to inoculate the production stage (modified Kannan and Rehacek media), which was incubated at 30 °C, 200 rpm for 48 h. The temperature was controlled at 30 °C, pH was set at 6.8 and 1 vvm air was supplied to the bioreactor. At the end of the fermentation, cells were retrieved by centrifugation. Wet biomass was homogenised and stored at −20 °C overnight, followed by lyophilisation. P(3HO-*co*-3HD) was produced by *P. mendocina* CH50 using glucose at 20 g/L concentration as the sole carbon substrate. Fermentation was carried out in two stages. The seed culture was prepared by inoculating sterile nutrient broth with a single colony of *P. mendocina* CH50. This was incubated for 16 h at 30 °C, 200 rpm. Then, 10% (*v*/*v*) of the inoculum was used to inoculate the second stage seed culture (mineral salt medium—MSM), which was incubated at 30 °C, 200 rpm for 24 h. Next, 10% (*v*/*v*) of the second stage seed culture was used to inoculate the final PHA production media (MSM media). The temperature was controlled at 30 °C, pH was set at 7 and 1 vvm air was supplied to the bioreactor. At the end of the fermentation, cells were retrieved by centrifugation. Wet biomass was homogenised and stored at −20 °C overnight followed by lyophilisation. PHA was extracted from the dried biomass using the Soxhlet extraction method. Methanol was used to remove the impurities from the biomass under reflux for 24 h. Methanol was then replaced with chloroform, and the biomass was subjected to Soxhlet extraction for another 24 h. The chloroform solution containing polymer was concentrated in a rotary evaporator. The PHA was precipitated using ice-cold methanol solution and stored at room temperature [70,71,72].

### 2.3. Gas Chromatography Mass Spectrometry (GC-MS)

The monomeric composition of the PHA produced was identified using GC-MS. Prior to the GC-MS analysis, polymer samples were methanolysed. GC-MS analysis was carried out using a Varian GC/MS system consisting of Chrompack CP-3800 gas chromatograph and Saturn 200 MS/MS block as described in Constantinides et al., 2018.

### 2.4. Production of the 3D PHA Scaffolds

The 3D PHA scaffolds were prepared using the particulate leaching technique. P(3HB) and P(3HO-*co*-3HD) were dissolved in chloroform, in a 50:50 ratio. Sodium chloride (<300 µm) was used as the porogen to create porous 3D PHA scaffolds. It was added to the polymer solution in a 1:9 (polymer:porogen) ratio and stirred for 24 h at room temperature to allow for homogeneous dispersion. The polymer solution containing porogen was poured into a Teflon mould (Dimensions—20 mm × 16 mm × 10 mm) and allowed to dry. The dry 3D PHA scaffolds were removed from the mould using a sterile scalpel and immersed in sterile water to allow porogen leaching. The pH of the sterile water containing 3D PHA scaffolds was measured to ensure complete removal of the porogen. Post leaching, the scaffolds were dried. They were visibly porous and were approximately 20 mm × 15 mm × 8 mm in size.

### 2.5. Cell Culture on 3D PHA Scaffolds

The 3D PHA scaffolds were cut into 10 mm × 10 mm × 8 mm using a sterile scalpel and placed in 24-well plates. Prior to seeding cells, the 3D PHA scaffolds were sterilised on both sides under high-intensity ultraviolet radiation, concentrated around a wavelength of 253.7 nm for 30 min. The 3D PHA scaffolds were further sterilised by washing them with 70% ethanol for 5 min. This was repeated thrice. Post sterilisation, they were allowed to dry for 12 h in the 37 °C humidified chamber to allow for ethanol to volatilise away. Sterile 3D PHA scaffolds were then rinsed thrice in 1× phosphate buffer saline (PBS) (Sigma Aldrich, St. Louis, MO, USA) and then incubated in 2 mL of Dulbecco’s modified Eagle medium (DMEM) supplemented with 2 mM L-glutamine, 10% FBS and 1% Penicillin-Streptomycin for the next 24 h.

HCT116 (ATCC^®^-CCL-247), MCF-7 (ATCC^®^-HTB-22) and MDA-MB-231 (ATCC^®^-HTB-26) cell lines were obtained from ATCC. The cells were cultured in T75 flasks (Sigma Aldrich) in DMEM (Himedia) containing 10% FBS (Thermo Fisher Scientific, Waltham, MA, USA), 2 mM L-Glutamine (Merck Life Science UK Limited, Dorset, UK) and 1% antibiotic solution (100 U/mL penicillin, 100 µg/mL streptomycin) (Sigma Aldrich), henceforth referred to as complete DMEM. Cultured cells were maintained at 37 °C, 5% CO_2_ in a humidified incubator. Static surface seeding method was used to seed cells onto the 3D PHA scaffolds as described in previous papers [73]. The ratio of the cell-seeded and scaffold area was optimised. Different cell seeding densities were used. The higher cell density of 200,000 cells/scaffold of 3 × 10 × 10 or 10 × 10 × 10 mm^3^ scaffolds was found to be optimal. Hence, a concentrated cell suspension of 200,000 cells was added to each 3D PHA scaffold and incubated for 30 min to allow for cells to attach to the scaffold. After 30 min, 500 µL (enough to cover the 3D PHA scaffold) of complete DMEM was carefully added from the sides so as to not dislodge any cells seeded on the top of the 3D PHA scaffolds and incubated for 12 to 24 h. After 12 h, 1000 µL of complete DMEM was added from the sides. At the end of incubation, the 3D PHA scaffolds were submerged in complete DMEM and covered with a cell crown 24 (Scaffdex Oy, Tampere, Finland). The cell-seeded 3D PHA scaffolds were incubated for 5 days. After every 24 h, media were aspirated, 3D PHA scaffolds were washed three times with PBS and complete DMEM was added to the 3D PHA scaffolds.

### 2.6. Cell Proliferation Studies

Proliferation of the cells on the 3D PHA scaffolds at the end of each time point was determined using the Alamar Blue assay following the manufacturer’s protocol (Thermo Fisher Scientific, Gloucester, UK). Alamar Blue reagent (10% volume of culture media) was added to the wells. After 3 h of incubation, Alamar Blue solution was transferred to a 96-well plate to obtain absorbance values at 570 nm. Tissue Culture Plastic (TCP) was used as the positive control.

### 2.7. Scanning Electron Microscopy (SEM)

#### 2.7.1. SEM Characterisation of the 3D PHA Scaffolds

Post leaching, dried 3D PHA scaffolds were observed under the Scanning Electron Microscope (SEM). The 3D PHA scaffolds were cut using a clean scalpel. The samples were vacuum-dried and placed on aluminium stubs. Finally, the 3D PHA scaffolds were gold coated and imaged using SEM Zeiss EVO40. The pore sizes were calculated from the SEM images using the Image J program (Public Domain Image Processing Program, National Institute of Health, Bethesda, MD, USA).

#### 2.7.2. SEM Characterisation of the Cell Laden 3D PHA Scaffolds

SEM was used to view the cross section of the cell laden 3D PHA scaffolds to observe cell morphology within the 3D PHA scaffold. Cell seeded 3D PHA scaffolds were fixed in 2% paraformaldehyde in PBS and kept at 4 °C overnight. They were dehydrated using graded ethanol solution (50%, 70%, 80%, 90% and 100%) and Hexamethyldisilazane (HMDS), and they were gold plated and imaged using SEM Zeiss EVO40 (Public Domain Image Processing Program, National Institute of Health, Bethesda, MD, USA).

### 2.8. Confocal Microscopy

The cell-seeded 3D PHA scaffolds cultured as described above were stained with CellTrace™ Calcein Green, AM/Ethidium homodimer (Invitrogen™)-1 mix in 1:4 ratio (MDA-MB-231) or 50 mM Image-iT TMRM reagent/10 µM CellTrace™ Calcein Green, AM (Invitrogen™) (HCT116 and MCF-7) for 30 min, cut using a clean scalpel into 1–2 mm slices placed in glass-bottom dishes and viewed using 488 nm and 548 nm wavelength, respectively, using a Nikon AIR microscope. In MDA-MB-231-seeded 3D PHA scaffolds, Calcein Green/Ethidium bromide led to green/red colour for live/dead analysis, whereas in HCT116- and MCF7-seeded 3D PHA scaffolds, TMRM/Calcein Green led to red/green colour, respectively. TMRM is a cell-permeant dye that accumulates in active mitochondria with intact membrane potentials indicating live cells.

### 2.9. Total RNA Extraction, cDNA Synthesis and qRT-PCR

Total RNA was extracted using Trizol (Sigma, Hertfordshire, UK), RNA concentration and purity was measured using the NanoDrop Spectrophotometer using absorbance values at 260 nm and 280 nm. cDNA was generated by the reverse transcriptase reaction and used for qPCR. The following genes were studied (corresponding primer sequences are given in references in parentheses): Wnt-11 [74]; Vim, Snail and E-cadherin [75]. Analysis by real-time qPCR was done by SYBR Green premix (Qiagen, Germantown, UK) using the following conditions: 95 °C for 15 min, 40 cycles at 95 °C for 15 s, 60 °C for 1 min and 72 °C for 15 sec. Relative levels of mRNA expression were calculated according to the CT/2-ΔΔCT method [64]. RNA polymerase II, (RPII) was optimised and used as the reference gene [76,77]. Experiments were performed in triplicate and the standard deviation was calculated as well as the Student’s *t*-test using GraphPad Prism 7.00 (La Jolla, CA, USA) software.

### 2.10. Data Analysis

All data were analysed as means ± standard errors. Statistical significance was determined using the student’s *t*-test or ANOVA with Newman–Keuls post hoc analysis, as appropriate. Results were considered significant for *p* < 0.05.

## 3. Results

### 3.1. Production and Chemical Characterisation of the Polymer to Make 3D PHA Scaffolds

Polymer production was carried out using bacterial fermentation. P(3HB) and P(3HO-*co*-3HD) polymers were produced using *Bacillus subtilis* OK2 and *Pseudomonas mendocina* CH50, respectively, with glucose as the sole carbon source as described in Basnett et al., 2021.

**Figure 1:**
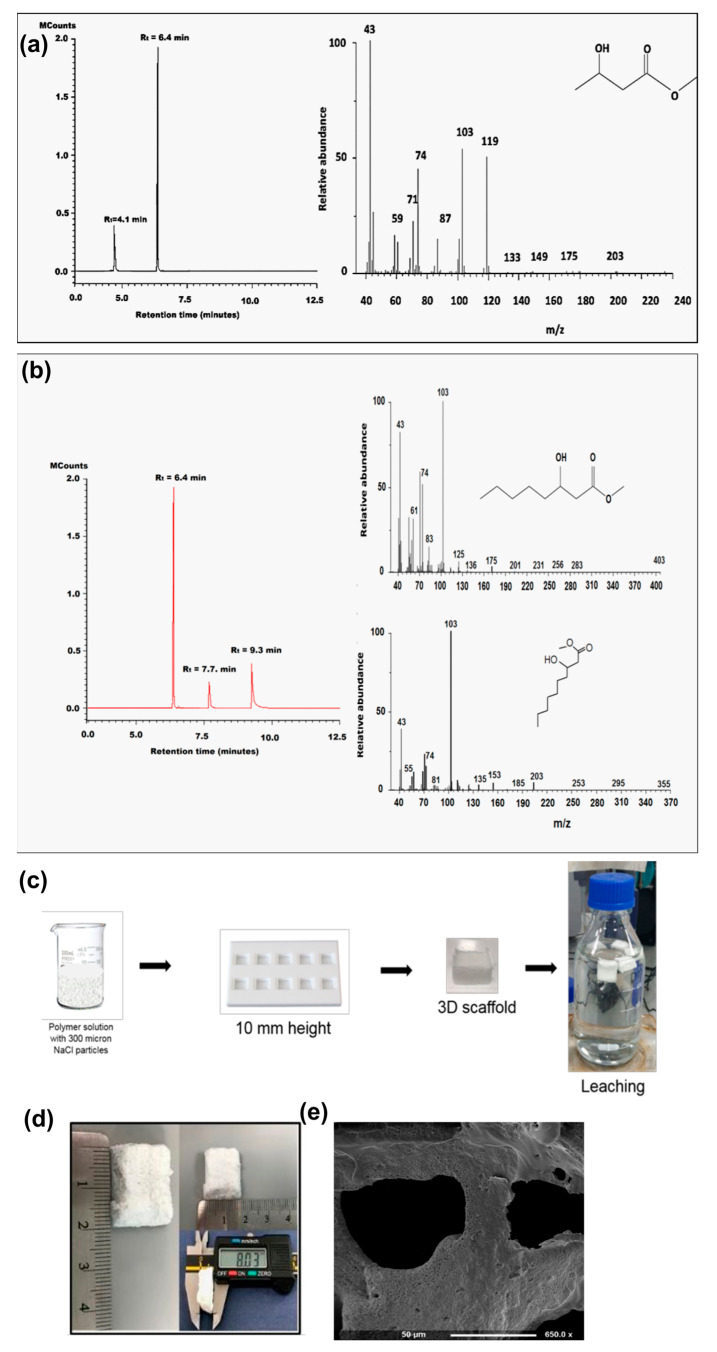
(**a**) GC-MS spectra of P(3HB) and (**b**) P(3HO-*co*-3HD). (**c**) Schematic representation of the preparation of porous 3D PHA scaffolds. (**d**) An optical image of air-dried porous 3D PHA scaffolds 20 mm × 15 mm × 8 mm. (**e**) SEM images of the P(3HB)/P(3HO-*co*-3HD) 50:50 3D PHA scaffold.

Gas Chromatography–Mass Spectrometry was used to confirm the monomeric composition of the PHAs produced. For P(3HB), the gas chromatogram showed one peak (R_t_ = 4.1 min), originating from the product of polymer methanolysis, and another peak of the internal standard, methyl benzoate (R_t_ = 6.4 min) (Figure 1a). Mass spectra pattern for the peak at R_t_ = 4.1 min matched with the methyl ester of 3-hydroxybutyric acid from the NIST Standard Reference Library. For P(3HO-*co*-3HD), the gas chromatogram showed two peaks (R_t_ = 7.7 min and R_t_ = 9.3). The mass spectra for the peaks matched with the methyl esters of 3-hydroxyoctanoic acid (3HO) and 3-hydroxydecanoic acid (3HD), respectively, from the NIST Standard Reference Library (Figure 1b).

In Figure 1a,b, the peak at 6.25 represented the internal standard (methyl benzoate). In the case of P(3HB), the fragment peak at retention time 4.125 represented the 3-hydroxy methyl ester of butyric acid, whereas in the P(3HO-*co*-3HD) spectrum, the fragment peak at retention time of 7.611 and 9.188 represented 3-hydroxyl-methyl ester of octanoic acid and decanoic acid, respectively.

### 3.2. Production and Characterisation of the 3D PHA Scaffold

The porous 3D PHA scaffolds were prepared by solvent casting–particulate leaching technique [67], which is a standard method to produce polymer-based scaffolds. The NaCl sieved through a 300 µm sieve ensured that the 3D PHA scaffolds have a well-connected variable pore size. The adoption of an appropriate polymer-to-salt ratio, experimentally defined as 1:9 for both P(3HO-*co*-3HD) and P(3HB), resulted in the formation of rich and interconnected porosity, and the choice of sieving salt through a 300 µm sieve allowed for controlling the final pore size. The 3D PHA scaffolds were soaked in distilled water to remove the porogen (Figure 1c). Stable pH of the water into which the porogen was being leached ensured the complete removal of the porogen, i.e., NaCl. The 3D PHA scaffolds appeared to be visibly porous without magnification. The 3D PHA scaffold size was 20 mm × 15 mm × 8 mm (Figure 1d). Larger 3D PHA scaffolds were produced, hoping to induce hypoxic conditions in the core.

Figure 1e shows SEM images of 3D PHA scaffolds, which were analysed further to analyse the structure and calculate the pore size of the 3D PHA scaffolds using Image J. The pore size varied from 30 to 300 µm, which provided a varied level of pore sizes within the 3D PHA scaffolds. The pores of variable sizes were evenly distributed throughout the 3D PHA scaffold, and an interconnected network of pores was observed.

### 3.3. Cell Culture on the 3D PHA Scaffolds to Create the Disease Models

#### 3.3.1. Hard Tumour Disease Models

**Figure 2:**
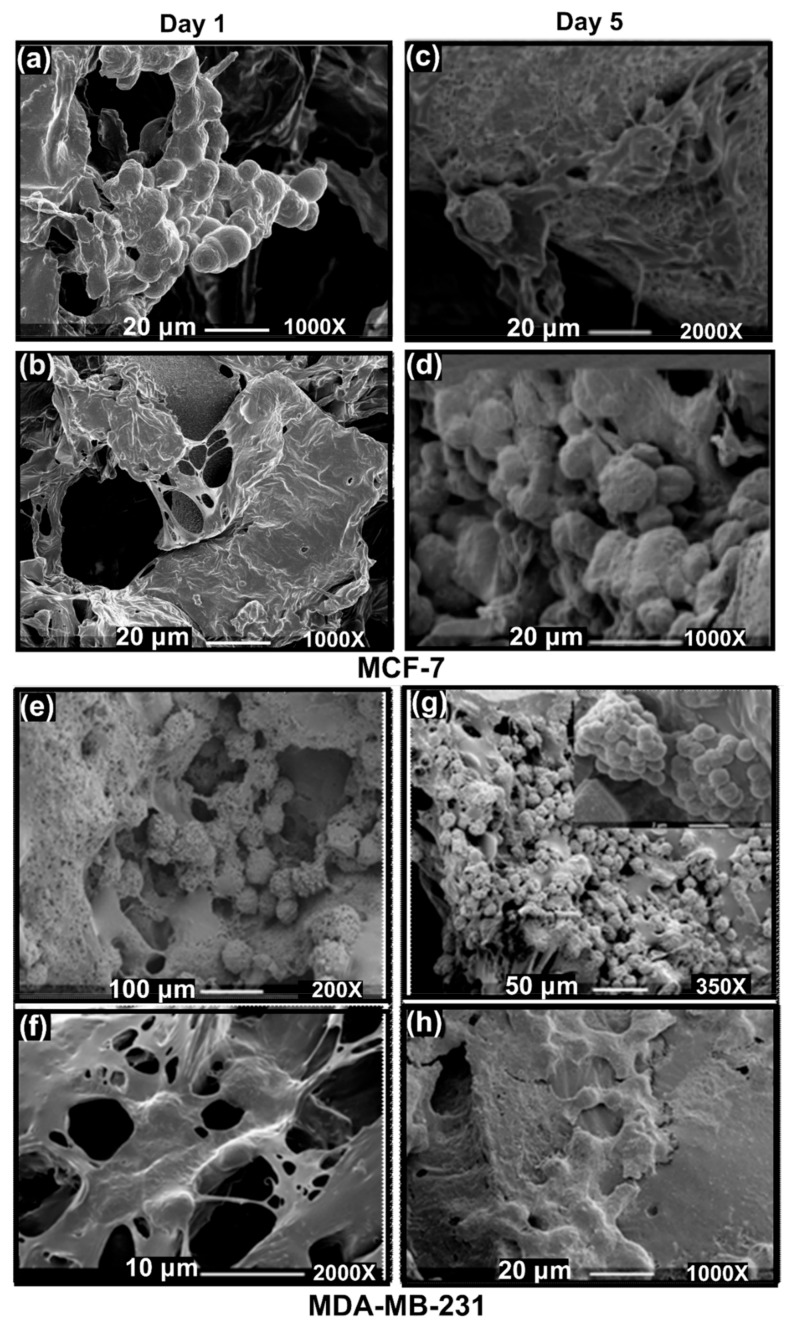
SEM images of cells grown on 10 mm × 10 mm × 8 mm PHA-based 3D scaffolds (**a**–**d**) MCF7 cells cultured on 3D PHA scaffolds at (**a**,**b**) Day 1 and (**c**,**d**) Day 5. (**b**) MDA-MB-231 cells cultured on 3D PHA scaffolds at (**e**,**f**) Day 1 and (**g**,**h**) Day 5.

**Figure 3:**
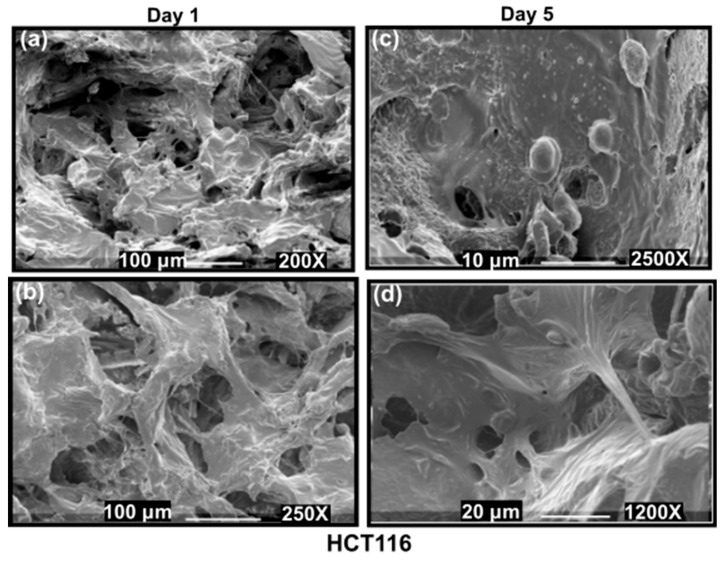
SEM images of HCT116 cells grown on 10 mm × 10 mm × 8 mm 3D PHA scaffolds on Day 1 (**a**,**b**) and Day 5 (**c**,**d**).

To show the ability of these 3D PHA scaffolds for development of tumour models two different human cancer cell lines derived from breast epithelium were chosen because of their different invasive properties and stiffness; basal MDA-MB-231 was derived from adenocarcinoma metastatic tumour site that is linked to aggressive disease, and MCF7 was derived from primary breast ductal carcinoma that belongs to the luminal A subtype [2,69]. These cell lines have significantly different stiffness. Several studies report that metastatic cells are softer as compared to their non-invasive counterparts [78,79]. Stiffness values of more aggressive cells (MDA-MB-231) are lower than their non-aggressive counterparts (MCF7) [80]. Each cell line was seeded on pre-soaked 3D PHA scaffolds and allowed to grow for 5 days. High cell seeding density per 3D PHA scaffold sample was chosen for cell viability studies to allow the cells to populate the 3D PHA scaffold, enabling cell–scaffold interaction as shown in Figure 2 and Figure 3.

#### 3.3.2. Cell Viability of Hard Cancer 3D Models

**Figure 4:**
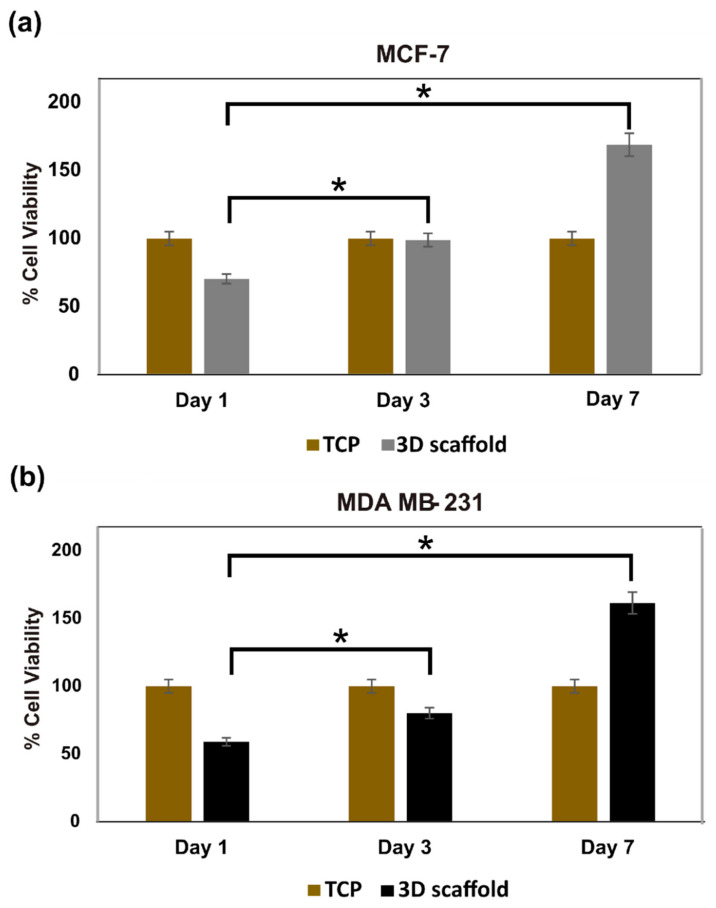
Alamar blue assay of (**a**) MCF-7 cells and (**b**) MDA-MB-231 cells cultured on 2D tissue culture plastic (TCP) and 3D PHA scaffold for 1, 3 and 7 days. Differences were considered statistically significant with *p* < 0.05 (*).

Viability of the cells on the 3D PHA scaffold was assessed on Day 1, 3 and 7 using the Alamar blue assay that detects metabolically active cells. The MCF-7 cells displayed a significant increase in cell viability with time (Figure 4a). On Day 1, 3 and 7, cell viability was set at 100% for the 2D cell culture on TCP. While at Day 1 on the 3D PHA scaffold, the cell viability was 74%, which significantly increased to 98.8% (*p* < 0.05) at Day 3. Again, on Day 7, there was a further significant increase in the cell viability of the MCF-7 cells to 168% (*p* < 0.05). Similarly, MDA-MB-231 cells displayed a significant increase in cell viability over time (Figure 4b). On Day 1, 3 and 7, the cell viability of the cells cultured on 2D cell culture on TCP were normalised to 100%. While the cells on the 3D PHA scaffolds showed only 59% cell viability on Day 1, which increased significantly to 80.2% on Day 3 (*p* < 0.05), followed by a further increase to 161.45% (*p* < 0.05) on Day 7.

These results show that cells in the 2D cell cultures exhibited higher proliferation rates than those in the 3D cultures initially, but the 3D cultures maintained a longer proliferation phase. This finding was consistent with previous studies [81].

#### 3.3.3. SEM Imaging of the Disease Models: Formation of Hard Cancer 3D Models

After 1 and 5 days of culture, cells were fixed on the 3D PHA scaffolds and observed under the SEM to assess their morphology. SEM images revealed that the MDA-MB-231 cells formed aggregates in contrast to the elongated shape observed when cultured on a 2D surface. The MCF-7 cells displayed diverse morphologies (Figure 2a,b,e,f). They proliferated into extensive layers of cells on the 3D PHA scaffold at Day 5. Hence, it seems like initially, on Day 1, the cells adhere to the 3D PHA scaffold and adjust to the new environment provided by the 3D PHA scaffold. However, by Day 5, the cells proliferate at a high rate, either forming layers covering the 3D PHA scaffold surface or growing in clumps where each cell surface could be viewed.

#### 3.3.4. Live Cell Assessment on the Hard Cancer Disease Models

**Figure 5:**
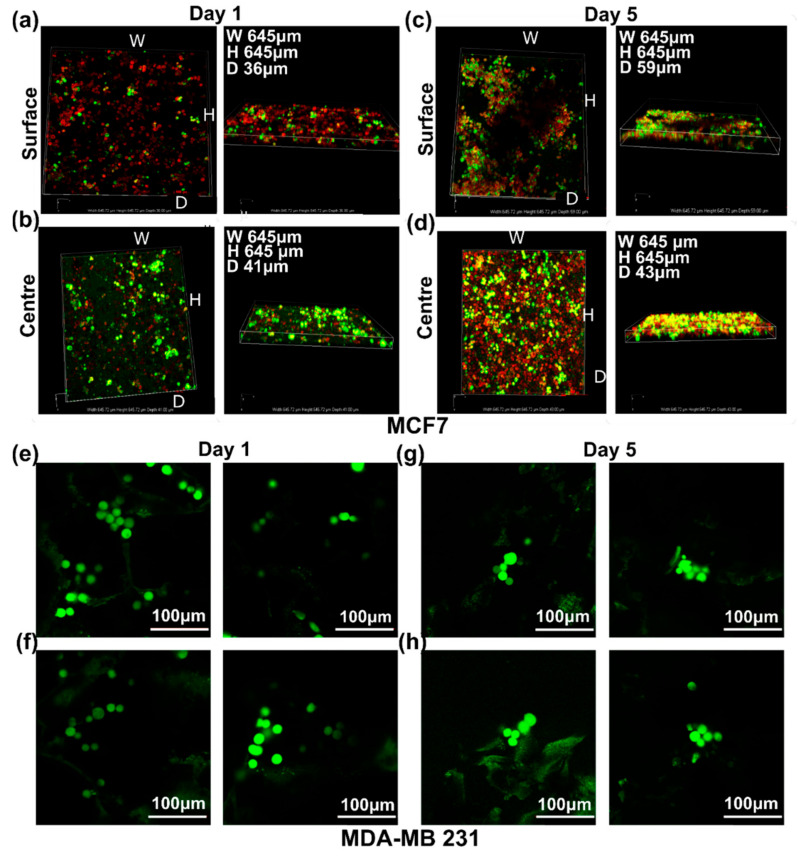
The 3D images of cells grown on 10 mm × 10 mm × 8 mm PHA-based 3D scaffolds (**a**–**d**). MCF7 cells cultured on 3D PHA scaffolds at (**a**,**b**) Day 1 and (**c**,**d**) Day 5 stained with TMRM (Red) and Calcein Green (Green), both for live cells. (**b**) MDA-MB-231 cells cultured on 3D PHA scaffolds at (**e**,**f**) Day 1 and (**g**,**h**) Day 5 stained with Calcein Green (Green) live cells and Ethidium bromide (Red) dead cells.

To investigate the growth pattern of breast cancer cell lines, MCF7 and MDA-MB-231 cells were grown on 10 mm 3D PHA scaffolds for a period of up to 5 days. The cells were stained with TMRM (Red) and Calcein green (green) to check the live cells within the 3D PHA scaffolds. TMRM detects active mitochondrial membrane and Calcein green detects live cells with intact cell membranes. On Day 1, the MCF7 cells were distributed evenly throughout the 3D PHA scaffold and no clumps were observed (Figure 5a,b). The cells spread throughout the centre and infiltrated to the bottom of the 3D PHA scaffolds (Figure 5b). On Day 5, MCF7 cells showed an even distribution throughout the 3D PHA scaffolds and there was no significant cell death, and they appear to be denser than Day 1 (Figure 5c,d). MCF7 cells formed an even dispersed layer on the 3D PHA scaffolds on Day 5, which covered most of the 3D PHA scaffold. The porosity of the 3D PHA scaffolds facilitates the cells to infiltrate to the bottom of the 3D PHA scaffolds, and a consistent distribution of cells were observed throughout the 3D PHA scaffold. Similar to the MCF7 cells, MDA-MB-231 cells also grew well on the 3D PHA scaffolds. Upon staining with Calcein Green (green), a lower density of MDA-MB-231 cells was observed on Day 1 (Figure 5e,f) and Day 5 (Figure 5g,h), and they tended to grow in clusters, as observed in native tumour tissue, throughout the 3D PHA scaffold. In comparison to MCF-7, there was not a marked difference in the growth pattern of MDA-MB-231 between Day 1 and Day 5.

#### 3.3.5. Wnt-11 and E-Cadherin mRNA Expression Profiling

Solid hard tumour models made by culturing breast cancer cell lines MCF7 and MDA-MB-231 were analysed for mRNA expression levels of several EMT marker genes. *Wnt-11*, *E-cadherin*, *Vim* and *Snail* genes were monitored over a period of 0, 7, and 14 days after seeding the cells in 3D PHA scaffolds. MCF7 and MDA-MB-231 cells were cultured in 3D PHA scaffolds, and cells were extracted from 3D PHA scaffolds on Day 0, 7 and 14 and analysed for mRNA levels.

**Figure 6:**
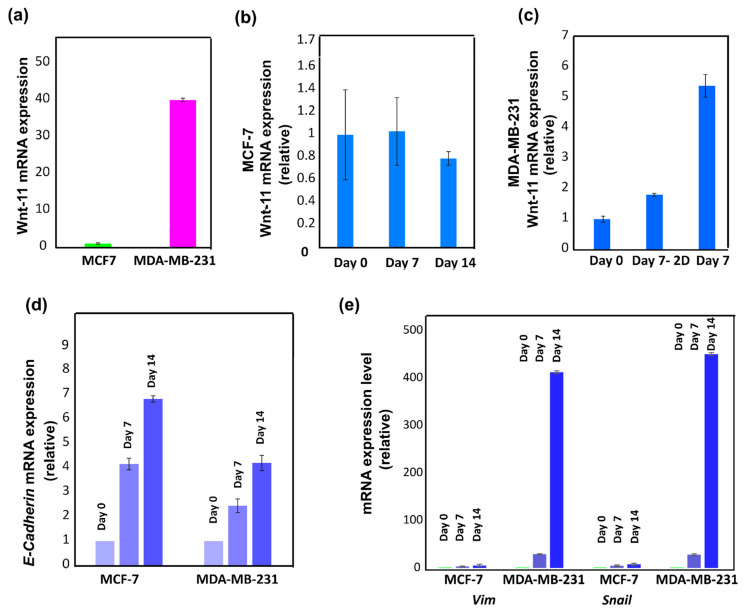
(**a**) qRT-PCR analysis of Wnt-11 expression level in MCF7 and MDA-MB-231 cells cultured in 3D PHA scaffolds. (**b**) Wnt-11 mRNA levels in MCF7 cells cultured in 3D PHA scaffolds for 0, 7 and 14 days. (**c**) Wnt-11 mRNA expression levels in MDA-MB-231 cells cultured on 3D PHA scaffolds for 0, 7 and 14 days. (**d**) E-Cadherin mRNA expression levels in MCF7 and MDA-MB-231 cells cultured in 3D PHA scaffolds for 0, 7 and 14 days. (**e**) mRNA levels of Vim and Snail in MCF7 and MDA-MB-231 cells grown in 3D PHA scaffolds. The column graphic represents the average of three replicates of mRNA isolated from each cell line. The data are normalised according to RPII expression level by fold analysis (*n* = 3; *p* < 0.01).

We first analysed *Wnt-11* mRNA levels in both MCF-7 and MDA MB-231 cells. *Wnt-11* mRNA expression in both breast cancer cell lines were quantified after growing cells on 3D PHA scaffolds on Day 0 using qRT-PCR. The MCF-7 cells, which have the most epithelial properties, expressed less *Wnt-11* mRNA than MDA-MB-231 cells (Figure 6a; Day 0; 40-fold ± 0.5, *n* = 3; *p* < 0.01). Next, MCF-7 cells were seeded onto 3D PHA scaffolds and allowed to grow for 14 days, and *Wnt-11* mRNA levels were analysed in a time dependent manner. qRT-PCR analysis revealed that *Wnt-11* gene expression in MCF7 cells did not significantly change when the cells were grown on the 3D PHA scaffolds for 7 and 14 days. Similarly, MDA-MB-231 cells were seeded on the 3D PHA scaffolds and allowed to proliferate for a period of 14 days. Interestingly, *Wnt-11* mRNA levels were found to be significantly upregulated in MDA-MB-231 cells grown on 3D PHA scaffolds on Day 7 and 14 by 5 ± 0.2 and 229 ± 0.8-fold in comparison to Day 0, respectively.

*E cadherin* is another EMT marker that is an intracellular adhesion protein and marker of epithelial characteristics [68]. To see the effects of PHA-based 3D disease models, MCF-7 and MDA-MB-231 cells were seeded on 3D PHA scaffolds and allowed to proliferate for a period of 0, 7 and 14 days. mRNA levels of the *E-Cadherin* gene were studied using qRT-PCR analysis. It was observed that the expression level of *E-cadherin* was significantly higher in MCF7 cells than MDA-MB-231 cells and increased further on the Days 7 and 14, as reported earlier [82]. *Wnt-11* and *E-cadherin* mRNA levels were found to be inversely correlated in MCF-7 and MDA-MB-231 cells (Figure 6d; *n* = 3; *p* < 0.05). Next, mesenchymal markers *Vim* and *Snail* mRNA levels were analysed in MCF-7 and MDA-MB-231 cells cultured on 3D PHA scaffolds. Cells were allowed to proliferate for 0, 7 and 14 days, then subjected to qRT-PCR analysis. It was observed that in MDA-MB-231 cells, the expression levels of *Vim* and *Snail* increased significantly by 429- and 450-fold, respectively, when the cells were grown for 14 days as compared to on Day 0 (*n* = 3; *p* < 0.01). Enhanced *Vim* and *Snail* gene expressions were also found in MCF-7 cells on Day 7 as compared to Day 0 (5- and 8-fold, respectively), but significant upregulation of both *Vim* and *Snail* mesenchymal markers was observed after 14 days with respect to Day 0 (Figure 6e, *n* = 3; *p* < 0.01).

In summary, all the results obtained are consistent with the fact that 3D cell culture using the 3D PHA scaffolds are superior substrates as mimics of the microenvironment of tumours as compared to 2D cell culture (data not shown) to reliably study gene expression profiles as well as cellular behaviour. The role of increased EMT markers and Wnt-11 in 3D need to be studied further in the future.

### 3.4. Cancer Disease Modelling for Soft Tumours

#### 3.4.1. SEM Imaging of the Disease Models: Formation of Soft Cancer 3D Models

Human adenocarcinoma colorectal cell line HCT116 was seeded on the fabricated 3D PHA scaffolds of 10 ×10 × 8 mm size and was allowed to proliferate for 5 days. The cell line proliferated into flat layered sheets on the 3D PHA scaffold on Day 1. HCT116 cells proliferated further and layers of cells forming colonies spread out within the crevices of the 3D PHA scaffolds by Day 5 (Figure 3c,d). Hence, it seems like initially, on Day 1, the cells adhere and adjust to the new environment provided by the 3D PHA scaffold. However, by Day 5, the cells proliferated at a high rate, either forming layers covering the 3D PHA scaffold surface or growing in clumps where each cell surface could be viewed.

#### 3.4.2. Live Cell Assessment on the Soft Cancer Disease Models

**Figure 7:**
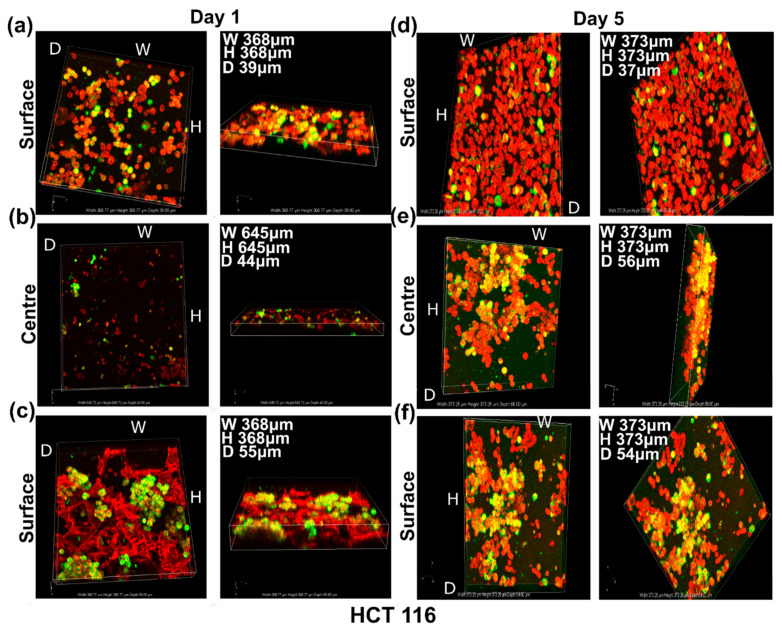
Live cell imaging of HCT116 cells grown on 10 mm × 10 mm × 8 mm 3D PHA scaffolds on Day 1—(**a**) upper side, (**b**) centre and (**c**) lower side—and on Day 5—(**d**) upper side, (**e**) centre and (**f**) lower side—using TMRM (Red) and Calcein Green (Green), both for live cells. The upper side, lower side and centre of the 3D PHA scaffold were imaged using a Nikon confocal microscope.

To make soft cancer models, HCT116 cells were allowed to grow in 3D PHA scaffolds for 5 days and imaged at Day 1 and 5 by staining them with TMRM (Red) and Calcein Green (Green). TMRM detects active mitochondrial membranes, and Calcein green detects live cells with intact cell membranes. HCT116 cells grow in pockets in the 3D PHA scaffolds rather than being dispersed throughout the 3D PHA scaffolds. This could be attributed to the fact that HCT116 grow in colonies in 2D cultures as well. By Day 5, HCT116 cells appeared to form bigger clusters (Figure 7a–c) than Day 1 (Figure 7d–f), and colonies started to appear in the pockets within the 3D PHA scaffolds with no significant cell death because the cells had enough time to adhere and adapt to the 3D PHA scaffold’s environment.

### 3.5. Comparison of Growth Patterns in Soft and Hard Cancer Disease Models

**Figure 8:**
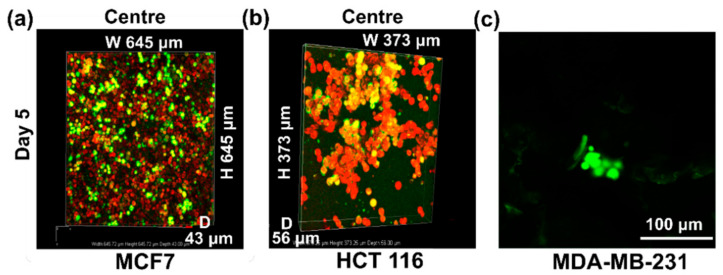
Comparison of cell growth patterns of (**a**) MCF7, (**b**) HCT-116 stained with TMRM (Red) and (**c**) MDA-MB-231 cells stained with Calcein Green (Green), both for live cells.

As seen in Figure 8, on Day 5, MDA-MB-231(representing hard breast tumour/cancer type) showed dispersed growth in clusters, and MCF7 cells (representing hard breast tumour/cancer type) formed an evenly dispersed dense layer, while HCT116 (representing soft colon tumour/cancer type) formed large colonies within the pockets of the 3D PHA scaffolds.

## 4. Discussion

In this study, an MCL and SCL-PHA blend (50:50 wt%) was used to fabricate a porous 3D PHA scaffold of size 10 mm × 10 mm × 8 mm and culture breast and colon cancer cells. There are many advantages of using PHAs. One main advantage of using PHAs is the fact that the mechanical properties of PHAs can be modulated to make models of variable stiffness. This is because there are many types of PHAs ranging from C_4_-C_16_ units in each monomer unit. By varying the carbon source provided to the bacteria and the bacterial species used, the monomer content of the PHA can be altered. This in turn leads to changes in the mechanical properties. The mechanical properties of the PHA-based 3D scaffolds can be tailored to match the specific tumour type by blending various types of PHAs or producing copolymers with different monomer types. This leads to an enormous range of mechanical properties that are not accessible for scaffolds using alginate/gelatine/hyaluronic acid or chitosan. In addition, PHA-based scaffolds have a slow degradation rate, and the degradation occurs by surface degradation. This results in stable scaffold structures, which can be used for long-term studies as opposed to the other types mentioned above. Additionally, PHAs are thermoplastics in nature and hence can be processed easily using a variety of processing methods, such as 3D printing using fused deposition modelling (FDM) and Selective Laser Sintering (SLS), to produce structurally varied and bespoke models.

Finally, and not the least, the size that can be achieved using PHA-based scaffolds is comparable to that of patient tumours. Since most tumours grow to a size of 1–2 cm when initially diagnosed, cancer cells grown in 3D PHA scaffolds of 1 cm thickness will resemble the oxygen gradient, nutrient and waste removal characteristics of in vivo tumours and emulate a tumour-like microenvironment [83,84]. Most 3D scaffolds that use biomaterials, such as gelatine, alginate and chitosan, have a lower size range, up to 600 µm [41,44,45]. 3D cell culture in the bigger 3D PHA scaffolds resembling tumour sizes is much more relevant to the understanding of cancer cell behaviour, identification of targets for cancer treatments and drug screening.

Hence, novel 3D cancer models were developed, where breast cancer and colon cancer cells were used to mimic hard and soft tumours, respectively. Breast tumours have higher stiffness as compared to colon tumours, which affects the oxygen gradient and nutrient supply in these tumours, and also affects the various cellular mechanisms [85]. PHAs are relatively slow in degradation and undergo surface degradation [86]. For substantial degradation, the scaffolds will need to be maintained in vitro for at least two to three months. In contrast, degradation of hydrogel-based scaffolds, such as for alginate and gelatine, occurs very fast. They undergo bulk degradation in less than 12 h in vitro [87]. It is extremely difficult to match the speed of hydrogel degradation with the pace of tissue formation, which is important in maintaining the shape and mechanical integrity of tissue-engineering constructs [10]. However, PHA-based scaffolds undergo surface degradation after 2–3 months as opposed to hydrogel scaffolds that would crumble and break during this period. Hence, PHA-based scaffolds remain stable for longer periods of time and can be used for long-term studies.

MCL and SCL-PHAs have been studied previously and have exhibited excellent biocompatibility with different types of cell lines for various applications including tissue engineering and medical devices [70,71,88]. However, MCL and SCL PHA blends have not been explored previously for their suitability as 3D PHA scaffolds in cancer disease modelling. The scaffold was developed using P(3HB), which is a short-chain length PHA, known to be hard and brittle in nature and P(3HO-*co*-3HD), which is a medium-chain length PHA, known to be soft and elastomeric. Therefore, a ratio of 50:50 was chosen as a first example to study the feasibility of the PHA-based models. In the future, the ambition is to use a range of the ratios and hence obtain scaffolds with a varying range of mechanical properties. The MCL and SCL-PHAs used for the fabrication of 3D PHA scaffolds were produced by the fermentation of *P. mendocina* CH50 and *Bacillus subtilis* OK2, respectively, using glucose as the carbon source. It is well established that *Pseudomonas* sp. are capable of accumulating MCL-PHA copolymers when grown on structurally unrelated carbohydrates [89,90]. Similarly, *Bacillus* species are known to produce the P(3HB) homopolymer when grown on glucose as substrate [91]. GC-MS was used to identify the MCL-PHA as a P(3HO-*co*-3HD) copolymer and the SCL-PHA as a P(3HB) homopolymer.

The P(3HO-*co*-3HD)/P(3HB) blend was used to fabricate 3D PHA scaffolds using the salt-leaching technique [92]. SEM images presented a foam-like structure with well-defined pores formed due to the dissolution of sodium chloride particles (Figure 1e).The pore size in the fabricated 3D PHA scaffolds ranged from 30 to 300 µm, the pores of variable sizes were evenly distributed throughout the 3D PHA scaffold, the interconnected network of pores facilitated the infiltration of cells throughout the 3D PHA scaffold and the cells had enough space to be able to grow in colonies and form 3D tumour models similar to the in vivo models. The pores would act as channels to facilitate cellular interaction, nutrient and oxygen diffusion, as well as waste removal [93,94]. Previously, various breast cancer cell lines from different subtypes, such as MCF-7 (luminal A), BT474 (luminal B), SKBR3 (human epidermal growth factor receptor 2—HER2) and MDA-MB-231 (triple negative), have been cultured in 3D microenvironments. The 3D liver model of Alginate (1% and 0.5%): GelMA (gelatine methacrylate) fibres containing NIH-3T3 fibroblasts, HepG2s and HUVECs showed no change in cell viability [41]. UV light illumination always carries the risk of high cell death and DNA damage, which affects normal cellular function. The Alginate:GelMA mixture was optimised for normal cellular function at a 365 nm wavelength [41]. This was not a concern in our study, as the 3D PHA scaffolds were UV sterilised prior to cell seeding. In this study, breast cancer cell lines, such as MCF-7 and MDA-MB-231, and the colon cancer cell line, HCT116, were cultured on the PHA-based porous 3D PHA scaffolds, and growth was observed for 1 and 5 days. Cancer cell properties, such as morphology, proliferation pattern and tumorigenicity, were monitored until Day 5. Both the cell lines attached and proliferated on the 3D PHA scaffold over time. In comparison to tissue culture plates, MCF-7 and MDA MB -231 cells cultured on 3D PHA scaffolds exhibited delayed growth until Day 3 (Figure 4). Both the cell types continued to proliferate on the 3D PHA scaffolds until Day 7, while cells cultured on 2D tissue culture plastic (TCP) displayed no change in cell viability on Day 7 (Figure 4). This could be due to the higher surface area of 3D scaffolds as compared to the 2D cell culture, which has a major drawback of contact inhibition to sustain cell growth over long durations [10]. It is the 3D environment in which the cells grow and maintain a longer proliferation phase, and are hence growing under conditions that mimic in vivo conditions [95].

These observations were consistent with a similar study conducted by Florczyk et al., 2016, where three cell lines, TRAMP-C2 (prostate cancer), SK-Hep-1 (liver cancer) and MDA-MB-231 (breast cancer), were cultured on 2D tissue culture plastic and Chitosan-Alginate 3D scaffolds [96].

It was observed that in the 2D cell culture, cell lines proliferate rapidly and become confluent, whereas cells show delayed growth, forming tumour spheres on the 3D scaffolds [96]. In another study conducted by Chen et al., 2012, MCF-7 cells were cultured on 3D collagen scaffolds. MCF-7 cells proliferated on the 3D PHA scaffolds until the Day 13, whereas these cells did not proliferate beyond Day 7 on 2D tissue culture plates [63]. This was concurrent with results obtained in our study, pointing out that cells cultured on the 3D PHA scaffolds initially take time to attach and start proliferation, unlike 2D cell culture, where cells soon fail to proliferate due to contact inhibition.

SEM images revealed that MDA-MB-231 cells formed aggregates and exhibited rounded morphology in a 3D environment while displaying a spindle-shaped morphology in the 2D cell culture. Similar observations were made by Ivers et al., 2014, when MDA-MB-231 cells were cultured in 3D, using a reconstituted basement membrane matrix Geltrex^®^ for 10 days. MDA-MB-231 form aggregates or spread in a dissociated manner showing elongated or round-shaped morphology, demonstrating the dynamic behaviour of the MDA-MB-231 in a 3D environment [97]. MCF-7 cells cultured on 3D PHA scaffolds displayed diverse morphologies in 3D (Figure 2a–d and Figure 5a–d). They proliferated into sheets with some rounded cells. While HCT116 formed large colonies within the pockets of the 3D PHA scaffold by Day 5, this needs further analysis, as they might make hypoxic pockets within the 3D PHA scaffold. This was consistent with the observation made by Chen et al., 2012. Do Amaral et al., 2011, made an observation that MCF7 cells formed unusual spheroids when cultured in 3D for longer periods of time [98]. This could explain the absence of grape like cell clusters on Day 5.

Live cell imaging of cells cultured on 3D PHA scaffolds provides a better analysis of the growth pattern and viability of hard and soft cancer cell lines. On Day 1, the MCF7 cells were distributed evenly throughout the 3D PHA scaffold, and no clumps were observed (Figure 5a,b). The cells spread throughout the centre and until the bottom of the 3D PHA scaffolds. While HCT116 cells grew in pockets rather than being dispersed throughout the 3D PHA scaffolds (Figure 6a–c). This could be attributed to the fact that HCT116 cells also grow in colonies in 2D cultures. On Day 5, MCF7 cells showed an even distribution throughout the 3D PHA scaffold, and there was no significant cell death and they appeared to be denser than Day 1 (Figure 5c,d). However, HCT116 cells appeared to form bigger clusters on Day 5 as compared to Day 1 (Figure 7d–f), and colonies started appearing in the pockets within the 3D PHA scaffolds with no significant cell death because the cells had sufficient time to adhere and adapt to the 3D PHA scaffold’s environment. Similarly, on Day 5, MCF7 cells formed an evenly dispersed layer, which covered most of the 3D PHA scaffold. HCT116 continued to form larger colonies within the pockets of the 3D PHA scaffold, which needs further analysis as they might make hypoxic pockets within the 3D PHA scaffold, as mentioned above. The porosity of the 3D PHA scaffolds facilitated the penetration of the cells to the bottom of the scaffolds and resulted in a consistent distribution of cells throughout the 3D PHA scaffold.

In chitosan alginate (CA) scaffolds, both hepatocellular carcinoma as well as human glioblastoma U-87 MG and U-118 MG cell lines showed increase in the expression levels of genes involved in EMT and cancer stem cells [99,100]. The mixed hydrogel of chitosan and hyaluronic acid (CH) used for human non-small cell lung cancer cells 3D spheroid formation showed increase in the expression level of EMT marker, stemness or drug resistance compared with those of cells in the 2D culture system [101]. 3D human glioblastoma cancer stem cells cultured in CH scaffolds also enhanced the expression of stem cell markers and drug resistance [102]. Gene expression profiles in hard cancer disease models were studied in a time-dependent manner. MCF7 and MDA-MB-231 cells cultured on 3D PHA scaffolds were analysed for mRNA expression levels of several EMT marker genes. *Wnt-11*, *E-cadherin*, *Vim* and *Snail* genes were monitored over a period of 14 days after seeding the cells in 3D PHA scaffolds. Our results demonstrated that MDA-MB-231 cells, when grown within 3D PHA scaffolds, express higher levels of *Wnt-11* and mesenchymal markers such as *Vim* and *Snail* mRNAs. Wnt signalling regulates a variety of cellular processes, including differentiation, cellular proliferation and stem cell pluripotency [74,103]. It has been reported that triple-negative breast cancer, which is an aggressive subtype of breast cancer, and expresses high levels of Wnt-11, which is accepted as a cancer stem cell (CSC)-like marker [60,61]. Epithelial cancer cells undergoing EMT adopt a cancer stem cell-like phenotype and are uniquely capable of seeding new tumours [104]. Moreover, it has been reported that TGFβ is linked to both EMT and Wnt-11. A recent study used TGF-β1 stimulation to investigate angiogenesis [105]. Future studies are required to determine the role of TGF-β1 in the PHA-based models.

E-cadherin is considered a pivotal marker in the EMT mechanism [106]; thus, we analysed the mRNA levels of *E-cadherin* in MCF7 and MDA-MB-231 cells as an “epithelial” marker at the molecular level. Both cell lines show increased *E cadherin* expression over a period of 14 days, which suggests gain of epithelial characteristics. This is inconsistent with the gain of EMT phenotypes as studies report *E-cadherin* to be linked to intercellular adhesion and to epithelial characteristics. However, most breast cancers are invasive ductal carcinoma and express *E-cadherin* in primary tumours and metastasis, which suggests that the PHA-based models resemble tumour expression profiles. We found increased *E-cadherin* mRNA levels in MCF-7 cells in a time-dependent manner, whereas MDA-MB-231 cells showed more ‘mesenchymal’ characteristics by increasing their *Snail* and *Vim* mRNA expressions. The complex genetic changes required to attain EMT-linked phenotypic changes are mediated by specific transcription factors including Snail (also known as Snail1). In 3D PHA scaffolds, the expression level of *E-cadherin* was significantly higher in MCF7 cells than MDA-MB-231 cells. Unlike MDA-MB-231 cells, MCF7 cell lines grown in 3D PHA scaffolds for 14 days show no significant change in *Vim* and *Snail*. This observation explains the significantly higher expression level of *E-cadherin* in MCF7 cells than MDA-MB-231 cells. The Snail acts as a transcriptional repressor of *E-cadherin* to regulate epithelial-mesenchymal transitions [107]. Snail l can be considered as one of the master EMT regulators and modulates cancer cell survival, cell cycle regulation, apoptosis evasion, cell adhesion, neuroendocrine differentiation and chemoresistance [106,108]. Snail also modulates the expression of a large number of genes directly or indirectly, associated with cancer invasion and metastasis to promote EMT in vitro [66,109]. Studies in tumour samples report that *Vim* is a downstream gene of Snail and is expressed by Snail to attain the EMT phenotype [107]. This explains why both breast cancer cell lines show a consistent change in their *Vim* and *Snail* mRNA levels, with increase in the transcription factor *Snail* gene expression and its downstream gene *vim* in MDA-MB-231, while in MCF7 cells, no change was observed in their expression. Studies of EMT markers in “basal-like” breast tumours reported that EMT markers (vimentin), as well as cadherin switching (reduced expression of *E-cadherin*), were significantly more frequent [110]. The epithelial components of breast carcinomas express *E- cadherin*, a proportion of them also show vimentin expression, while the mesenchymal components of breast carcinomas show vim expression [110].

These observations point out the resemblance in the EMT markers in breast cancer cell lines cultured in 3D PHA scaffolds. The EMT gene expression profiles of MCF7 and MDA-MB-231 cells cultured in 3D PHA scaffolds were similar to those seen in tumour samples, which is representative of better cancer models that can be further analysed for their tumour-like characteristics. The PHA-based cancer models result in better stemness characteristics and molecular marker resemblance to tumours. Our data confirmed that PHA-based 3D scaffolds allowed for breast cancer cells to grow in 3D, and the EMT/Wnt11 gene expressions increased significantly as shown by others in Matrigel-based 3D cultures. In summary, all the results are consistent with the fact that PHA-based 3D disease models are comparable to other biomaterials to study gene expression profiles as well as cellular behaviour as compared to 2D cell culture. In addition, in future, the 3D model developed in this work could be used to enhance the percentage of tumour development in vivo, especially with cells from patients, a truly bespoke model.

## 5. Conclusions

This is the first ever study investigating the suitability of 3D PHA scaffolds for the development of cancer models using breast cancer and colon cancer cell lines. We produced two types of PHAs, P(3HB) and P(3HO-*co*-3HD), using *Bacillus subtilis* and *Pseudomonas mendocina*, respectively, and with good yields. These were blended to form high-quality 3D scaffolds with controlled interconnected porosity. Three types of cancer cell lines, MCF7 cells, MDA-MB-231 (breast cancer cells) and HCT116 (colon cancer cells), were successfully grown within the PHA-based 3D scaffolds, exhibiting excellent proliferation and cellular morphology mimicking that of native cancer cells. These results confirmed that the PHA-based 3D scaffolds provided a suitable 3D environment for the cancer cells and would work very well to create functional 3D disease models to be used for in-depth understanding of the process of cancer development and for novel drug testing. Both Wnt-11 and EMT have been linked to the stem cell phenotype. These results could contribute to an understanding the cellular behaviour of cancer cells and help in finding better targeted therapies. Therefore, the role of increased EMT markers and Wnt-11 in cancer cells grown within the 3D disease models needs to be further explored. These models will be helpful in analysing the gene expression, cellular signalling pathways, angiogenesis and chemotherapy response more accurately than 2D and other currently available 3D models.

## Data Availability

All the data can be accessed in the main text.
